# Beliefs and barriers associated with COVID-19 vaccination among the general population in Saudi Arabia

**DOI:** 10.1186/s12889-021-11501-5

**Published:** 2021-07-21

**Authors:** Rania M. Magadmi, Fatemah O. Kamel

**Affiliations:** Pharmacology Department, Faculty of Medicine, King Abdulaziz University, P.O. Box 42751, Jeddah, 21551 Saudi Arabia

**Keywords:** COVID-19, COVID-19 vaccine acceptance, Saudi Arabia, Vaccine, Vaccine hesitancy

## Abstract

**Background:**

Developing a vaccine against COVID-19 is considered a key strategy to end the pandemic. However, public acceptance is reliant on beliefs and perception toward the vaccine. Therefore, the study aimed to assess the beliefs and barriers associated with COVID-19 vaccination among the Saudi population.

**Methods:**

An online self-administered questionnaire was distributed across the main regions of Saudi Arabia on May 2020. The questionnaire addressed the socio-demographic variables, beliefs toward COVID-19 vaccination, and potential barriers that may prevent participants from being vaccinated. The association between COVID-19 vaccine acceptance and sociodemographic variables were analyzed. Logistic regression analysis was used to identify the predicting variables of vaccine acceptance.

**Results:**

Out of 3101 participants, 44.7% were accepting of COVID-19 vaccination if available, whereas 55.3% admitted hesitancy. Younger, male, who received seasonal influenza vaccine were more likely to accept taking the vaccine. The study found that concerns about side effects were the key barrier for vaccine acceptance. Furthermore, the majority of refusers may accept the vaccine if additional studies confirmed safety and effectiveness.

**Conclusion:**

Results can be utilized in planning vaccination campaigns while waiting for vaccine development.

**Supplementary Information:**

The online version contains supplementary material available at 10.1186/s12889-021-11501-5.

## Background

The coronavirus disease-19 (COVID-19) is highly contagious and caused by severe acute respiratory syndrome coronavirus 2 (SARS-CoV-2) [1]. On 30 January 2020, the World Health Organization (WHO) declared an outbreak of a public health emergency of international concern [2]. During the first 6 months, more than 10 million COVID-19 cases were confirmed worldwide, out of which more than 20 thousand were in Saudi Arabia [3]. Vaccination is considered the most effective strategy for preventing the pandemic and avoiding complications associated with the disease [4]. Two COVID-19 vaccines have approved in Saudi Arabia (Pfizer/BioNTech, Oxford-AstraZeneca) recently [5]. However, many studies [6, 7] have shown that the decision to take available vaccines is dependent on beliefs and perceptions. Therefore, worldwide concern regarding public acceptance of an eventual vaccine for COVID-19 has been increasing [8, 9].

A recently published review [10] demonstrated that vaccine acceptance and hesitancy vary at the global context. In Saudi Arabia, a COVID-19 vaccine is expected to face significant public hesitancy given the current public hesitancy toward seasonal influenza vaccination [11, 12]. Thus, the study aimed to assess the beliefs of Saudi residents toward eventual COVID-19 vaccination and to uncover the barriers associated with vaccination among the general population in Saudi Arabia.

## Methods

### Study design

The study is cross-sectional in nature and was conducted on May 2020. A validated, self-administered electronic questionnaire was distributed online through social network sites, such as WhatsApp and Twitter.

### Participants

The study targeted potential participants from the main five regions in Saudi Arabia to attain results that would be generalizable across the country. The questionnaire has been designed and developed for the purpose of this study (supplement 1). At the beginning of the questionnaire, the participants were briefly informed about the objective of the study and the different sections of the questionnaire. Prior to the main questionnaire, the participants were asked three screening questions to ensure that they meet the inclusion criteria of the purposive sampling strategy (i.e., above 18-year old, a resident of Saudi Arabia, and agreeable to sharing responses).

### Questionnaire

A pilot study was carried out on 30 participants to assess the face validity of the Arabic and English versions of the questionnaire. Furthermore, a pretesting of the both versions of the questionnaire was conducted to assess the content validity. Two research epidemiological experts independently reviewed all questionnaire items. Their feedbacks were considered in the final version of the questionnaire. The final questionnaire consisted of three domains, where the questions aimed to collect data on socio-demographic variables, beliefs toward COVID-19 vaccination, and potential barriers that may prevent participants from being vaccinated, respectively.

The questionnaire was distributed in Arabic and English languages. Translation was performed using the backward–forward method and double-checked by the authors.

### Ethical considerations

The study was approved by the Unit of Biomedical Ethics Research Committee at the Faculty of Medicine, King Abdulaziz University (Reference No. 275–20) dated May 19, 2020.

### Statistical analysis

Data cleaning and analysis were performed using the Statistical Package for Social Sciences (SPSS) v. 24.0 (IBM Corporation, Armonk, New York, USA). Descriptive statistics including frequencies (n) and percentage (%) were used to present the demographic data of the participants.

For the second domain, responses were rated from 0 (upper-most limit of negative belief; anti-vaccination) to 3 (upper-most limit of positive belief; pro-vaccination). The points from each question were summarized to calculate the total score. A total score of > 2 was considered positive belief, whereas the opposite is true for a total score of < 2.

Chi-square test was used to assess the significance of the association (contingency) between COVID-19 vaccine acceptance and sociodemographic variables. Logistic regression analysis was used to calculate the odds ratio (OR) and 95% confidence intervals (95% CI) to identify the predicting variables of vaccine acceptance. A *P*-value < .05 was considered statistically significant.

## Results

### Demographic characteristics

A total of 3101 participants from five regions in Saudi Arabia were recruited. Table 1 shows that more than half of the participants (53.4%) were aged 40–59 years, 58.3% were female, and the majority (89.7%) were Saudi. More than two-third (63.9%) obtained a university degree. Other participants were engaged in jobs with high risk of COVID-19 infection (healthcare workers = 13.3%; food-related professions, such as catering = 0.6%, interaction with foreigners = 2.2%). The most and least numbers of participants were derived from the Western and Northern regions, respectively. Only 25% declared a history of chronic disease. Lastly, approximately 40% were definite about obtaining the seasonal influenza vaccine.

**Table 1:** Sociodemographic characteristics of participants (*n* = 3101)

Characteristics	Frequency (n)	Percentage (%)
Age		
18–29	390	12.6%
30–39	781	25.2%
40–59	1657	53.4%
> 60	273	8.8%
Sex		
Men	1294	41.7%
Women	1807	58.3%
Nationality		
Saudi	2783	89.7%
Non-Saudi	318	10.3%
Educational level		
Primary	6	0.2%
Elementary	24	0.8%
Secondary	300	9.7%
University	1983	63.9%
Higher education	788	25.4%
Occupation		
Healthcare workers	411	13.3%
Catering	19	0.6%
Working with foreigners	68	2.2%
Other jobs	1592	51.3%
Unemployed (including retirees, students, and housewives)	1011	32.6%
Region		
Western	1928	62.2%
Central	394	12.7%
Eastern	377	12.2%
Northern	101	3.3%
Southern	301	9.7%
Do you have any chronic disease?		
Yes	786	25.3%
No	2315	74.7%
Did you get the seasonal influenza vaccine before?		
Yes	1366	44.1%
No	1411	45.5%
Not sure	324	10.4%

### Beliefs toward COVID-19 vaccination

Table 2 illustrates uncertainty among the participants regarding the safety (55.4%) and effectiveness (56.1%) of eventual COVID-19 vaccination whenever available. However, the majority agreed that getting the vaccine is the best means of avoiding the complications of COVID-19 (46%). In summary, only one-third of the participants showed positive beliefs toward COVID-19 vaccination.

**Table 2:** Participants’ beliefs toward safety of COVID-19 vaccination (*n* = 3101)

Beliefs toward COVID-19 vaccination	All subjects n (%)
Do you think that COVID-19 vaccination, whenever available, would be safe?	
Yes	886 (28.6%)
No	497 (16%)
Not sure	1718 (55.4%)
Do you think that COVID-19 vaccination, whenever available, would be effective?	
Yes	1066 (34.4%)
No	294 (9.5%)
Not sure	1741 (56.1%)
Do you think that the best way to avoid the complications of COVID-19 is by being vaccinated?	
Yes	1428 (46%)
No	867 (28%)
Not sure	806 (26%)
If COVID-19 vaccination is available, are you planning to get it?	
Yes	1386 (44.7%)
No	1715 (55.3%)
Total score for beliefs	
Positive	1021 (32.92%)
Negative	2080 (67.08%)

The same pattern of beliefs was found among a high-risk group who was defined on the following criteria: > 60 years old, healthcare worker, working with foreigners, working in catering, or having chronic diseases (supplement 2).

### COVID-19 vaccine acceptance

Table 3 shows the characteristic of individuals who would accept and refuse COVID-19 vaccination. Out of 3101 participants, 44.7% declared that they are planning to be vaccinated. Participants aged less than 30 years were found to be 1.572 times more likely to accept vaccination (95% CI: 1.06–2.33) compared with those aged > 60 years. Similarly, participants with secondary or university education were 1.75 (95% CI: 1.26–2.44) and 1.295 (95% CI: 1.06–1.59) times more likely to accept vaccination, respectively. Moreover, the male participants were 1.204 times likely to accept vaccination (95% CI: 1.01–1.44).

**Table 3:** Predicting factors of COVID-19 vaccine acceptance

Variable	Planned to obtain COVID-19 Vaccination		OR	95% CI	*P*
	Yes (%)	No (%)			
Total	*n* = 1386 (44.7%)	*n* = 1715 (55.3%)			
Age					
18–29	238 (61%)	152 (39%)	1.57	1.06–2.33	.024*
30–39	351 (44.9%)	430 (55.1%)	0.9	0.63–1.29	.566
40–59	696 (42%)	961 (58%)	1.01	0.76–1.47	.736
> 60	101 (37%)	172 (63%)	–	–	–
Gender					
Male	626 (48.4%)	668 (51.6%)	1.2	1.01–1.44	.04*
Female	760 (42.1%)	1047 (57.9%)	–	–	
Nationality					
Saudi	1241 (44.6%)	1542 (55.4%)	0.94	0.72–1.24	.67
Non-Saudi	145 (45.6%)	173 (54.4%)	–	–	.
Educational level					
Primary	4 (66.7%)	2 (33.3%)	2.89	0.46–18.24	.260
Elementary	12 (50%)	12 (50%)	2.19	0.85–5.62	.104
Secondary	154 (51.3%)	146 (48.7%)	1.75	1.26–2.44	.001*
University	899 (45.3%)	1084 (54.7%)	1.3	1.06–1.59	.014*
Higher education	317 (40.2%)	471 (59.8%)	–	–	
Occupation					
Healthcare workers	194 (47.2%)	217 (52.8%)	1.36	0.17–1.69	.285
Catering	6 (31.6%)	13 (68.4%)	0.73	0.21–2.6	.627
Other jobs	708 (44.5%)	884 (55.5%)	1.15	0.61–2.17	.670
Unemployed	450 (44.5%)	561 (55.5%)	0.99	0.52–1.89	.977
Working with foreigners	28 (41.2%)	40 (58.8%)	–	–	
Region					
Western	845 (43.8%)	1083 (56.2%)	–	–	–
Central	165 (41.9%)	229 (58.1%)	0.84	0.64–1.09	.187
Eastern	173 (45.9%)	204 (54.1%)	1.04	0.79–1.37	.76
Northern	60 (59.4%)	41 (40.6%)	1.47	0.91–2.39	.116
Southern	143 (47.5%)	158 (52.5%)	1.15	0.86–1.53	.357
Do you have any chronic disease?					
Yes	346 (44%)	440 (56%)	0.95	0.78–1.17	.549
No	1040 (44.9%)	1275 (55.1%)	–	–	–
Did you get the seasonal influenza vaccine before?					
Yes	700 (51.2%)	666 (48.8%)	1.59	1.19–2.14	.002*
No	532 (37.7%)	879 (62.3%)	1.02	0.76–1.37	.892
Not sure	154 (47.5%)	170 (52.5%)	–	–	–
Beliefs					
Positive (*n* = 1021)	806 (78.9%)	215 (21.1%)	9.29	7.7–11.2	.000*
Negative (*n* = 2080)	580 (27.9%)	1500 (72.1%)	–	–	–

As expected, participants with a history of taking previous seasonal influenza vaccines were 1.594 times more likely to accept COVID-19 vaccination (95% CI: 1.19–2.14). A remarkable increase in the likelihood of being vaccinated was observed for those who held positive beliefs. In this case, the participants with positive beliefs toward COVID-19 vaccination were 9.288 times more likely to accept vaccination if available (95% CI: 7.72–11.17).

However, Table 3 illustrates that nationalities, occupations, chronic diseases, or residence in regions across Saudi Arabia were unable to predict vaccination behavior (*P*-value > 0.05).

### Barriers associated with COVID-19 vaccination

Table 4 shows the barriers associated with acceptance of COVID-19 vaccination. More than one answer was available for each item; hence, cumulative percentages exceeded 100%. The majority of vaccine refusers were concerned about side effects (80%). Approximately 25% lack confidence in the effectiveness of vaccination (23.4%). One-fifth of the precipitants supported the conspiracy theory surrounding COVID-19, whereas the remainder believed that vaccines are unnecessary because they are strongly compliant with personal hygiene practices and social distancing or because they consider themselves healthy and not at risk.

**Table 4:** Participants’ barriers associated with acceptance of COVID-19 vaccination

Barriers	Vaccine refusers (*n* = 1715)
I am concerned about the vaccine’s side effects.	1371 (79.9%)
I don’t believe that the vaccine will stop the infection.	401 (23.4%)
COVID-19 vaccination is a conspiracy.	380 (22.2%)
I don’t need the vaccine because I do all the right things. I wash my hands and wear a mask and gloves.	372 (21.7%)
I don’t need the vaccine because I’m young and healthy.	176 (10.3%)
I don’t like needles.	38 (2.2%)
Other	275 (16%)
Options to encourage future COVID-19 vaccination	
If my physician recommended it to me	353 (20.6%)
If I know that more studies showed that the vaccine is safe and effective	1096 (63.9%)
If it was compulsory by the government (MOH)	754 (44%)
If it was mandatory by my job	203 (11.8%)
If my family or friends got vaccinated	102 (5.9%)
If there is a way other than injection	71 (4.1%)
I would not take it in anyway.	305 (17.8%)
Other	71 (4.1%)

However, more than two-third of vaccine refusers (63.9%) indicated that they will agree to be vaccinated if further studies confirmed the safety and effectiveness of COVID-19 vaccination. A total of 44% of vaccine refusers will agree to vaccination if made compulsory by the government but only 11% if made compulsory by their jobs not the government. Furthermore, one-fifth of vaccine refusers may accept vaccination if recommended by physicians. A significant percentage of vaccine refusers (17.8%) will not take the vaccine under any of the cited circumstances.

## Discussion

Results indicated that the sample population was divided between vaccine acceptance and refusal whenever available. This finding highlights the dilemma of the topic in the Saudi population, where half of the population may accept vaccination. Moreover, the study revealed several key predictors of hesitancy toward COVID-19 vaccination. Older females with high levels of education, no history of influenza vaccine uptake, and negative beliefs toward vaccination were more likely to display hesitation toward COVID-19 vaccination. Notably, being part of a high risk group did not improve the odds of being vaccinated. The most significant predictor of vaccination is holding positive beliefs.

Although studies that assessed hesitancy toward COVID-19 vaccination are limited, the acceptance/ hesitancy rates toward any vaccine are diverse across the world [10]. Correspondingly, the percentage of hesitancy toward COVID-19 vaccination in the present study was twice that of the percentage reported for China [8], the USA [13], and Egypt [14]. This could be explained as a result of the impact of the multicultural society in Saudi Arabia, impact of rumours and incomplete information spread through social media channels.

The most significant predictor for acceptance of COVID-19 vaccination was beliefs. This result is congruent with that of a previous systematic review conducted by Bish et al. [15] to assess the evidence for factors associated with H1N1 vaccine acceptance. The authors reported that participants’ beliefs toward H1N1 vaccination was strongly associated with the intention to be vaccinated in studies carried out in Turkey, Australia, the United Kingdom, and Malaysia [15].

Results demonstrated that self-reported influenza vaccine uptake was a positive predictor for the acceptance of eventual COVID-19 vaccination. Similarly, previous studies have shown that the rate of H1N1 vaccine acceptance was higher among participants with a history of uptake of the seasonal influenza vaccine in the USA [16] and France [17]. Of concern, however, the self-reported rate of influenza vaccine is very low across regions in Saudi Arabia. A study carried out in the Western region of Saudi Arabia indicated that only 18.5% of people received the influenza vaccine in 2015 [18]. The same rate was reported in the Central region of Saudi Arabia in 2011 [19]. Given that COVID-19 is highly contagious with high mortality rates, a significant portion of the population should be vaccinated for the prevention of the disease. However, given the correlation between previous influenza vaccine use and likelihood of accepting COVID-19 vaccination, the current study argued that the Saudi population may experience low rates of vaccination whenever COVID-19 vaccination becomes available.

Male gender was another positive predictor for acceptance of COVID-19 vaccination. This result could be due to the reported high rates of COVID-19-related morbidity and motility among male infected patients [20]. Furthermore, women tend to support COVID-19 conspiracy theories at a higher proportion than men [21], which may be one of the factors that can explain women’s higher resistance to vaccination.

Another positive predictor for the acceptance of COVID-19 vaccination was age. Younger participants tended to be more accepting of vaccination in contrast with older participants. The same trend was observed among participants with secondary and university levels of education compared to participants with higher education. A possible explanation is that younger participants are more frustrated with social restrictions and curfews associated with the COVID-19 crisis and would thus be more willing to be vaccinated. At the same time, younger people may be more accustomed and trusting of science and technology in contrast with their older counterparts. At the same time, school suspension may negatively affect the academic performance of school-aged and university participants. Therefore, they are more impatient to bring an end to the situation and thus more accepting of vaccination. Further studies should explore these possibilities as these data can be useful for future vaccination campaigns.

Although a previous study indicated that the majority of Chinese healthcare workers were willing to accept COVID-19 vaccination [8], more than half of Saudi healthcare workers in this study displayed hesitancy toward vaccination. However, hesitancy toward influenza vaccination has been previously reported among Irish [22] and Saudi [23] healthcare workers. At the other end of the spectrum, Dempsey et al. [24] underlined the positive influence of healthcare professionals on increasing the uptake of human papillomavirus vaccination among adolescents in a randomized clinical trial. Given these aspects, the finding of the present study regarding the hesitancy of healthcare workers in Saudi Arabia toward COVID-19 vaccination is concerning for the following reasons. First, healthcare workers are at high risk of COVID-19 infection and thus of spreading the disease. Secondly, healthcare workers play a central role in convincing people to be vaccinated. This role will most likely be pivotal in increasing the uptake of COVID-19 vaccination. This tendency indicates that future research should focus on assessing the scale of reluctance toward vaccination among Saudi healthcare workers and on developing and testing interventions that may improve vaccination uptake rates and beliefs among healthcare workers in Saudi Arabia.

As stated by MacDonald [25], factors influencing hesitancy toward vaccination could be related to confidence, complacency, and/or convenience. In the present study, lack of confidence in the safety and effectiveness of vaccination were the main barriers preventing the acceptance of COVID-19 vaccination among the population. The speedy pattern of the development of the prospective COVID-19 vaccines could be one of the reasons behind the lack confidence in vaccination, which is similar to reports on the H1N1 pandemic [26].

Another barrier to COVID-19 vaccination was the COVID-19 conspiracy theory, which has spread very rapidly around the world [9, 27, 28] via social media platforms, precisely where the study participants were recruited. This scenario may indicate that future studies should use a different sampling population such as probability sampling with proper stratification.

The widespread conspiracy theory could be due to the people’s psychological need to understand the unexpected events associated with the COVID-19 pandemic [29]. Moreover, the conspiracy theory has been reported as a factor for hesitancy toward vaccination, such as that during the H1N1 pandemic [30] and influenza vaccine among the Saudi population [23].

In agreement with previous research in China [8], the majority of vaccine refusers stated that they require additional research to confirm the safety and effectiveness of vaccination before acceptance. This finding could be explained partially by the fact that majority of the participants in the current study were at the university level. Moreover, the majority of vaccine refusers achieved higher levels of education. Consequently, their background knowledge may contribute to their judgment on the vaccination concept.

Notably, during the H1N1 influenza A pandemic, the public acceptance rate of Americans toward vaccination before its approval was 8.7% [31]. However, the rate of self-reported vaccination uptake increased to 20% after a vaccine was introduced to the market [32].

Given that vaccination is the cornerstone of reduced healthcare burden caused by the COVID-19 pandemic, the results of the study can be utilized for planning evidence-based vaccination campaigns while waiting for vaccine development [33]. By enhancing people’s beliefs over vaccination and by understanding the barriers to acceptance of COVID-19 vaccination will most likely enhance people’s acceptance, which may result in a maximized vaccine uptake when it becomes available.

The current study has certain limitations. The study was conducted using an online self-administered questionnaire instead of face-to-face interviews due to the implemented curfew and social distancing restrictions during the COVID-19 pandemic. As a result, reporting bias should be considered. Moreover, the cross-sectional study represents public acceptance and beliefs toward COVID-19 vaccination during the pandemic before the availability of a vaccine. With this notion, people’s acceptance and beliefs could be changed with time as reported in other studies on pandemics [16].

Furthermore, disparities were noted across regions with regard to response rate. The highest response rate was noted for the Western region, which is one of the largest in the country. Makkah, AlMadinah, and Jeddah cities can be found in this region, which are the most affected cities by COVID-19 in Saudi Arabia. This scenario could make people from the region more aware and anxious about topics related to COVID-19. Other factors may have contributed, such as availability of the Internet as well as differences in perception toward the use of social media platforms across regions.

## Conclusion

Results from current study can be utilized in planning vaccination campaigns to increase the public awareness.

## Supplementary Information

**Additional file 1: Supplement 1.** English version of the questionnaire. **Supplement 2.** Barriers associated with acceptance of COVID-19 vaccine among high risk group (*N* = 882).

## Data Availability

Data that support the findings in the current study are available from the corresponding author on reasonable request.

## Abbreviation

COVID-19: Coronavirus disease-19
CI: Confidence intervals
MOH: Ministry of health
OR: Odds ratio
SARS-CoV-2: Severe acute respiratory syndrome coronavirus 2
WHO: World Health Organization
