# Supplementary material for: Beliefs and barriers associated with COVID-19 vaccination among the general population in Saudi Arabia
Source: BMC Public Health. 2021 Jul 21;21:1438. doi: 10.1186/s12889-021-11501-5 (PMC8294288; doi:10.1186/s12889-021-11501-5)
Supplement: Supplementary file 1 — Additional file 1: Supplement 1. English version of the questionnaire. Supplement 2. Barriers associated with acceptance of COVID-19 vaccine among high risk group (N = 882). [file 12889_2021_11501_MOESM1_ESM.docx]

**Beliefs and barriers associated with COVID-19 vaccination among the general population in Saudi Arabia**

**Rania M. Magadmi^a*^, Fatemah O. Kamel^a^**

**Supplement 1. English version of the questionnaire**

**Part 1. Demographic Data**

|  | Age: | 18-29 | 30-39 | 40-59 | | ≥60 | | |  | | |  |  |  |
| --- | --- | --- | --- | --- | --- | --- | --- | --- | --- | --- | --- | --- | --- | --- |
|  | Gender: | Male | Female |  | |  | | |  | | |  |  |  |
|  | Nationality: | Saudi | Non-Saudi |  | |  | | |  | | |  |  |  |
|  | Education level: | Non-educated | Elementary | Secondary | | University | | | Higher education | | |  |  |  |
|  | Occupation: | Healthcare staff | Jobs dealing with food (Catering) | other jobs. | | Not worker (retired, student, housewife). | | | working with foreigners | | |  |  |  |
|  | Do you have any chronic disease ? | Yes | No |  | |  | | |  | | |  |  |  |
|  | Did you get the seasonal influenza vaccine before? | Yes | No | Not sure | |  | | |  | | |  |  |  |
|  |  |  |  | |  | |  | |  | |  |  |  |  |
|  | **Part II:** **Beliefs toward COVID-19 vaccination** | | | | | | | | | | | | | |
|  | Do you think that the COVID-19 vaccine, whenever available, would be safe? | Yes | No | | Not sure | | |  |  | | | |  |  |
|  | Do you think that the COVID-19 vaccine, whenever available, would be effective? | Yes | No | | Not sure | | |  |  | | | |  |  |
|  | Do you think that the best way to avoid the complications of COVID-19 is by getting the vaccine? | Yes | No | | Not sure | | |  |  | | | |  |  |
|  | Do you think that greater public awareness is needed about COVID-19  vaccine? | Yes | No | |  | | |  |  | | | |  |  |
| 1. 1 | If the COVID-19 vaccine is available, are you planning to get it? if “Yes” , please stop here. and if “No” , please answer the following questions. | Yes | No | |  | | |  |  | | | | |  |
|  | **Part III: Barriers associated with COVID-19 vaccination** | | | | | | | | | | | | | |
|  | Check which statement show why you do not plan to get COVID-19 vaccine (check all that apply). | I am concerned about the vaccine’s side effects. | I don’t believe that the vaccine will stop the infection. | | I don’t need the vaccine because I do all the right things. I wash my hands and wear a mask and gloves. | | | I don’t like needles. | The COVID-19 vaccine is a conspiracy. |  |  |  |  |  |
|  |  | I don’t need the vaccine because I’m young and healthy. | Other…… | |  | | |  |  |  |  |  |  |  |
|  | Under those scenarios, would you be more likely to get the COVID-19 vaccine? | If my physician recommended it to me. | If it was mandatory by my Job. | | If it was compulsory by the government (MOH). | | | If my family or friends got vaccinated. | If I know that more studies showed that the vaccine is safe and effective. |  |  |  |  |  |
|  |  |  | I would not take it in any  situation. | | If there is a way other  than injection. | | | Other…….. | |  |  |  |  |  |

**Supplement 2. Barriers associated with acceptance of COVID-19 vaccine among high risk group (N = 882)**

| **Barriers** | **High risk group**  **N (%)** |
| --- | --- |
| I am concerned about the vaccine’s side effects. | 555 (62.9%) |
| I don’t believe that the vaccine will stop the infection. | 174 (19.7%) |
| The COVID-19 vaccine is a conspiracy. | 159 (18%) |
| I don’t need the vaccine because I do all the right things. I wash my hands and wear a mask and gloves. | 162 (18.4%) |
| I don’t need the vaccine because I’m young and healthy. | 57 (6.5%) |
| I don’t like needles | 15 (1.7%) |
| Other | 134 (15.2%) |
| **Options to encourage future COVID-19 vaccination** | |
| If my physician recommended it to me. | 148 (16.8%) |
| If I know that more studies showed that the vaccine is safe and effective. | 469 (53.2) |
| If it was compulsory by the government (MOH). | 292 (33.1) |
| If it was mandatory by my Job. | 95 (10.8) |
| If my family or friends got vaccinated. | 30 (3.4) |
| If there is a way other than injection | 30 (3.4) |
| I would not take it in anyway. | 122 (13.8) |
| Other | 23 (2.6) |
